# 850. Real-world effectiveness and safety compared among isavuconazole, amphotericin B, and voriconazole in invasive mold disease patients

**DOI:** 10.1093/ofid/ofad500.895

**Published:** 2023-11-27

**Authors:** Ye Qiu, Wushu Chen, Yangqing Zhan, Shaoqiang Li, Yan Wang, Zhen Chen, Zhengtu Li, Feng Ye

**Affiliations:** The First Affiliated Hospital of Guangzhou Medical University, Guangzhou, Guangdong, China; The First Affiliated Hospital of Guangzhou Medical University, Guangzhou, Guangdong, China; The First Affiliated Hospital of Guangzhou Medical University, Guangzhou, Guangdong, China; The First Affiliated Hospital of Guangzhou Medical University, Guangzhou, Guangdong, China; The First Affiliated Hospital of Guangzhou Medical University, Guangzhou, Guangdong, China; The First Affiliated Hospital of Guangzhou Medical University, Guangzhou, Guangdong, China; The First Affiliated Hospital of Guangzhou Medical University, Guangzhou, Guangdong, China; The First Affiliated Hospital of Guangzhou Medical University, Guangzhou, Guangdong, China

## Abstract

**Background:**

Isavuconazole is a brand-new triazole with broad-spectrum antifungal action. Amphotericin B and voriconazole are critical components of the antifungal arsenal. But the effectiveness and safety compared among these three drugs in invasive mold disease (IMD) are still lacking.

**Methods:**

Real-world study of effectiveness and safety compared among isavuconazole, amphotericin B, and voriconazole in IMD patients was performed between October 2019 and March 2022 in the First Affiliated Hospital of Guangzhou Medical University. We included the IMD patients who were treated with isavuconazole or amphotericin B or voriconazole. We tested the non-inferiority of the primary efficacy endpoint of all-cause mortality from the first dose of the study drug to day 42 in patients who received at least one dose of the study drugs using a 10% non-inferiority margin. Meanwhile, safety was assessed in patients who received the first dose of the study drugs.

**Results:**

236 patients with IMD including 33 patients in the isavuconazole group, 24 patients in the amphotericin B group, and 179 patients in the voriconazole group were included in our study(**Table 1**, **Figure 1**). Mortality up until day 42 was 6% in the isavuconazole group, 33% in the amphotericin B group, and 16% in the voriconazole group (I: Isavuconazole, A: Amphotericin B, V: Voriconazole; I vs A: treatment difference 27.3% [95% CI 6.6 to 47.9%]; p=0·0002. I vs V: treatment difference 10.1% [95% CI –5.3 to 25.6%]; p=0·0053. V vs A: treatment difference 17.1% [95% CI 3.17 to 31.1%]; p< 0·0001) (**Table 2, Figure 3**). We got differences among these three groups in renal and urinary disorders(RAUD)(P=0.015), hypokalemic blood(p=0.028), and systemic events(SE)(p=0.003). Patients who received amphotericin B typically experienced a higher rate of treatment-related adverse events; however, patients who had lower adverse events rates of SE (7%) received voriconazole, and of RAUD (3%), liver disorders (3%) received isavuconazole. (**Figure 2**).

**Figure 1.**
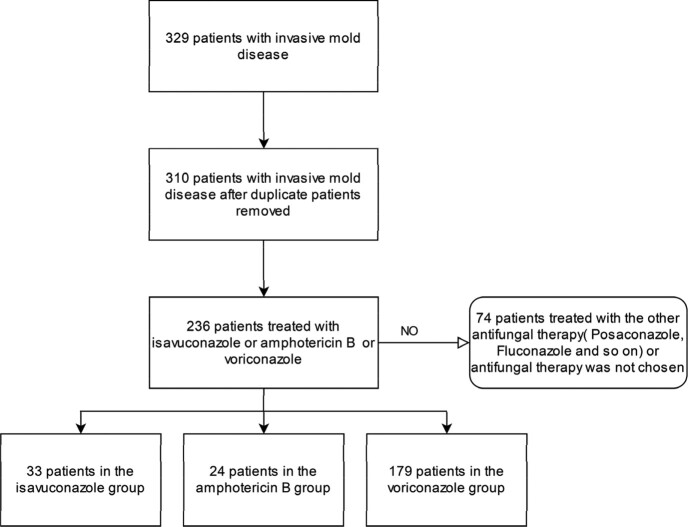
Patient Population

**Figure 2:**
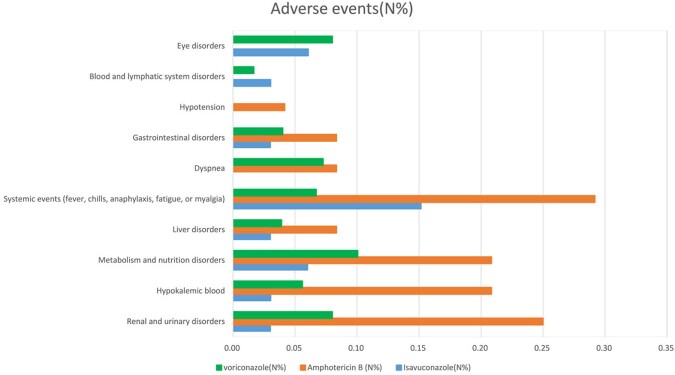
Adverse Events. Blood and lymphatic system disorders: thrombocytopenia, eosinophilia, paroxysmal nocturnal hemoglobinuria, and so on. Gastrointestinal disorders: nausea, vomiting, indigestion, abdominal pain, or diarrhea. Metabolism and nutrition disorders: hypoglycemia, hypoproteinemia, worsening adrenal insufficiency, or metabolic acidosis. Liver disorders: defined as elevated aspartate aminotransferase [AST] or alanine aminotransferase [ALT] three or more times the upper limit of normal, elevated total bilirubin two or more times the upper limit of normal, and alkaline phosphatase less than two times the upper limit of normal. Renal and urinary disorders: Ccr<70 ml/min or GFR <1/2 of the normal value or Cr>200μmol/L or SCr increased ≥ 26.5µmol/L within 48 hours or increase in SCr ≥ 1.5 times baseline in the past 7 days or Urine volume within 6 hours < 0.5mL/kg/hr.

**Figure 3:**
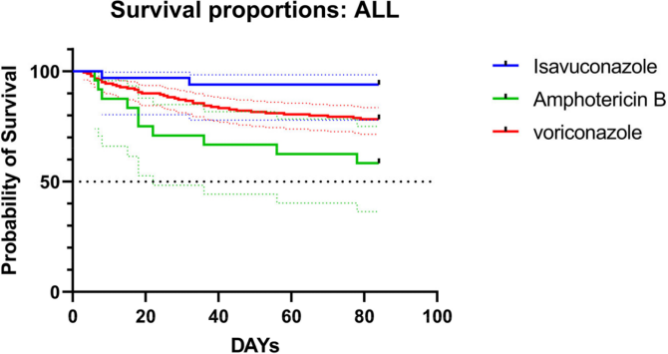
Survival from first dose of study drug to day 84 for all groups

**Conclusion:**

Isavuconazole and voriconazole were superior to Amphotericin B, and Isavuconazole was non-inferior to voriconazole, for the primary efficacy endpoint in patients with IMD. The results indicated that we need to attach importance to the role of isavuconazole so that we can better use isavuconazole in clinical practice.

Demographics and baseline characteristics

**Table 1:**
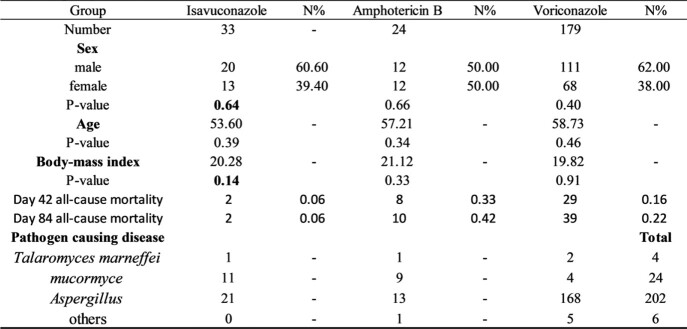
Demographics and baseline characteristics

Non-inferiority test

**Table 2:**
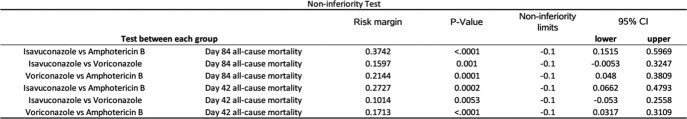
Non-inferiority test

**Disclosures:**

**All Authors**: No reported disclosures

